# Clinical Therapy of Metastatic Spinal Tumors

**DOI:** 10.3389/fsurg.2021.626873

**Published:** 2021-04-15

**Authors:** Jie Li, Wenjie Wei, Feng Xu, Yuanyi Wang, Yadong Liu, Changfeng Fu

**Affiliations:** ^1^Department of Spine Surgery, The First Hospital of Jilin University, Changchun, China; ^2^Key Laboratory of Polymer Ecomaterials, Changchun Institute of Applied Chemistry, Chinese Academy of Sciences, Changchun, China; ^3^Key Laboratory of Pathobiology, Ministry of Education, School of Basic Medical Sciences, Jilin University, Changchun, China

**Keywords:** metastatic spinal tumor, surgical management, minimally invasive therapy, radiation therapy, systemic therapy

## Abstract

Metastatic spinal tumors (MST) have high rates of morbidity and mortality. MST can destroy the vertebral body or compress the nerve roots, resulting in an increased risk of pathological fractures and intractable pain. Here, we elaborately reviewed the currently available therapeutic options for MST according to the following four aspects: surgical management, minimally invasive therapy (MIT), radiation therapy, and systemic therapy. In particular, these aspects were classified and introduced to show their developmental process, clinical effects, advantages, and current limitations. Furthermore, with the improvement of treatment concepts and techniques, we discovered the prevalent trend toward the use of radiation therapy and MIT in clinic therapies. Finally, the future directions of these treatment options were discussed. We hoped that along with future advances and study will lead to the improvement of living standard and present status of treatment in patients with MST.

## Introduction

### Epidemiology

The spine—as part of the skeletal system—is the most frequent site of metastatic tumors (1). The thoracic spine is the most commonly involved segment, followed by the lumbar spine and cervical spine (2). Most of the pathologic vertebral fractures occur in the T6–L4, followed by T11–L1. Of note, fractures are less frequent in the cervical spine (3). Metastatic spinal tumors (MST) are classified into three categories: intramedullary, intradural-extramedullary, and extradural tumors, according to their anatomical location. Extradural tumors account for 90–95% of MST (4). The highest known incidence of MST is reported in patients aged 40–65 years. This observation may be related to the high risk of cancer during this age (5). Various types of primary cancers frequently metastasize to the spine, including breast (21%), lung (14%), prostate (8%), and kidney (5%) (6). Histopathological studies indicated that the incidence of MST in patients with advanced cancer was 30–90%. Notably, the occurrence of bone metastases increases in parallel with the observed increase in patient survival (7).

### Clinical Symptoms and the Therapeutic Principle for MST

As stated earlier in this article, MST mainly occurs in the extradural compartment (8). These tumors result in a series of clinical symptoms, markedly impairing patient quality of life (9). Firstly, pain is a prominent feature of spine tumors, because of the elongated spine and expansion of the spinal epidural venous plexus (10). Pain of various degrees is reported in the vast majority of patients (≤ 90%) (11). Secondly, the development of MST results in the compression of neural structures, generating motor, sensory, and sphincter malfunction (10, 11). The average survival of patients with MST is 7 months, whereas that of patients with epidural spread is 3–6 months (12). This short life expectancy results in that the patients are suitable for regimens of palliative treatment rather than antitumor therapy. The primary goal of treatment against MST is to restore or preserve nerve function, relieve pain, and maintain or enhance the stability of the spine.

Therefore, the treatment decision making play a significant role in the therapy of MST. NOMS framework, the modified tokuhashi scores, the SINS scores and the tomita scores are all well-established and frequently used in establish prognosis.

The NOMS decision framework includes the neurologic, oncologic, mechanical, and systemic considerations. Besides, conventional external beam radiation, spinal stereotactic radiosurgery, minimally invasive and open surgical interventions also involved (13, 14). In short, neurological considerations are the evaluation of the degree of epidural spinal cord compression, myelopathy, or functional radiculopathy. Oncology considerations are based on the expected tumor response and the durability of the response to existing treatments. Mechanical instability is a separate consideration for pathological fractures and the considerations of treatment. The systemic considerations need to evaluate the tolerance of patients and the overall expected patient survival based on the degree of the disease and the malignancy of the tumor to the treatment method based on the systemic diseases (15).

The NOMS paradigm integrates multimodality therapy to form a decision-making framework that incorporates sentinel decision points into the therapy of MST. Taking into account the tumor sensitivity to radiation and the extent of epidural extension, the best radiotherapy and surgical decompression needs can be determined. The mechanical stability of the spine and the consideration of systemic diseases further help determine the necessity and feasibility of surgical intervention (13, 14). Moreover, other scores for evaluating the prognostic efficacy of metastatic spine disease include the modified tokuhashi scores, the SINS scores and the tomita scores (16). Overall, these four methods play important roles in the treatment decision making of MST. The flexible choice of these methods is the key to a good prognosis for patients (17).

**Figure 1:**
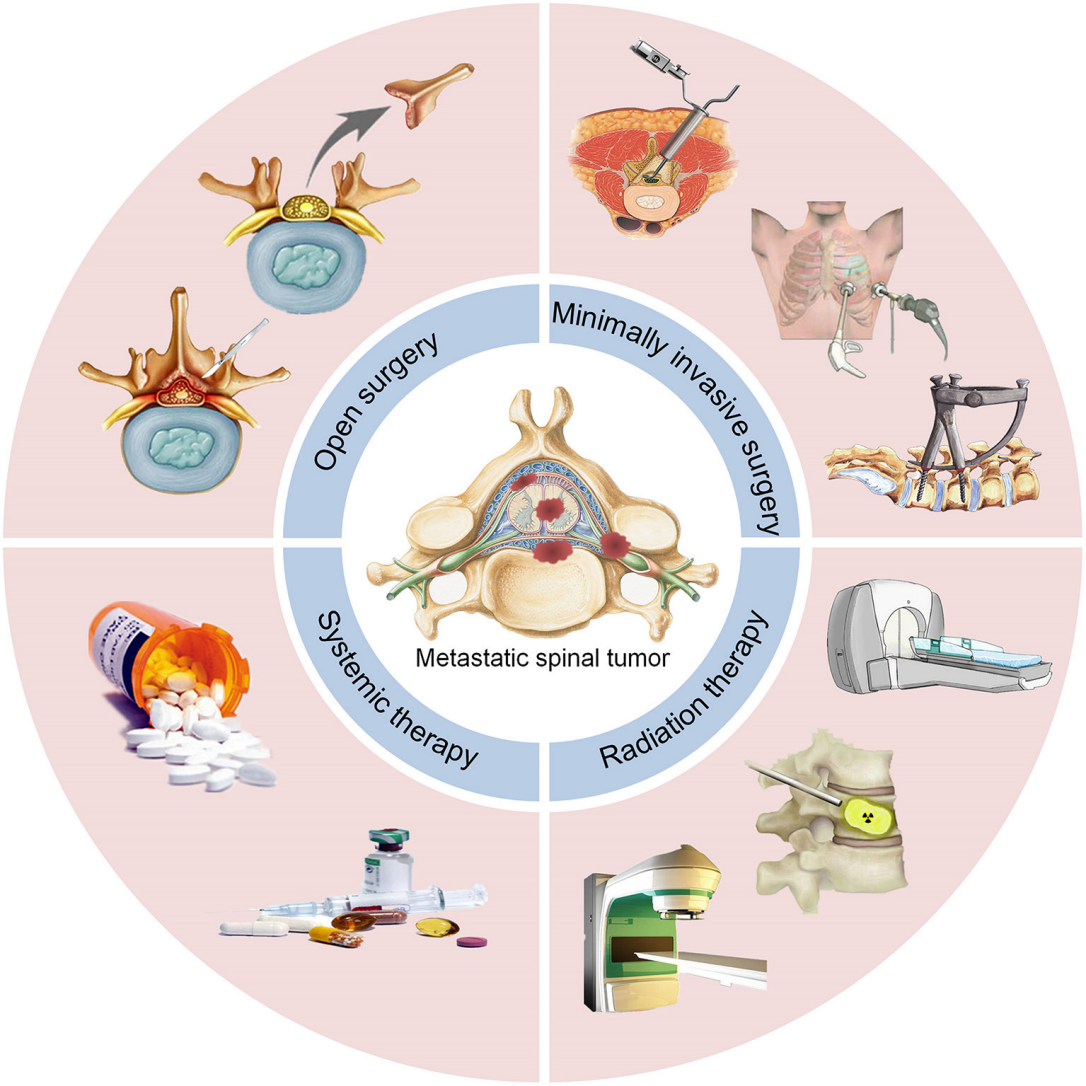
Location and current treatment options for MST. MST, metastatic spinal tumors.

In this systematic review, we recapitulate the currently available treatment options and advances in surgical management, minimally invasive therapy (MIT), radiation therapy, and systemic therapy for the management of MST patients (Figure 1).

## Surgical Management

Surgical treatment still plays a significant role in patients with intractable pain, progressive neurological damage, instability of the spine, failure of radiotherapy and chemotherapy, and high possibility of cure (18). Here we introduce four surgical methods, including open surgery, minimal access spinal surgery (MASS), percutaneous pedicle screw fixation (PPSF), video-assisted thoracoscopic surgery (VATS). The choice of surgical methods should follow the principle of individualized treatment and attach importance to the quality of life of patients.

### Open Surgery

Prior to the introduction of radiation therapy, laminectomy was the most commonly used method for spinal decompression. Through posterior approach laminectomy, decompression of the spinal cord theoretically reduces stress on the spinal cord and relieves nerve damage. The most frequently affected sites on account of MST are the vertebral bodies and pedicles (19). Therefore, the removal of these spine attachments further impairs the stability of the spine, resulting in poor surgical outcome. Notably, this outcome may be even worse than the efficacy observed after conservative treatment. Thus, open surgery was widely abandoned and radiation therapy became the preferred treatment option for MST (12, 20). The subsequent development of surgical techniques and modified instrumentation for spinal decompression improved the limitations of open surgery, providing a stable spine. Furthermore, the development of anterior approaches to the spine has enabled substantial tumor debunking from the vertebral body, leading to improved neurological outcomes.

Research performed by Patchell et al. (21) demonstrated a clear advantage of surgical treatment in remaining or even regaining ambulatory of patients. At present, surgical intervention remains the first therapeutic choice for many patients with spinal epidural metastases. Furthermore, the combination of surgery with radiation therapy significantly improves the prognosis of patients.

### Video-Assisted Thoracoscopic Surgery

**Figure 2:**
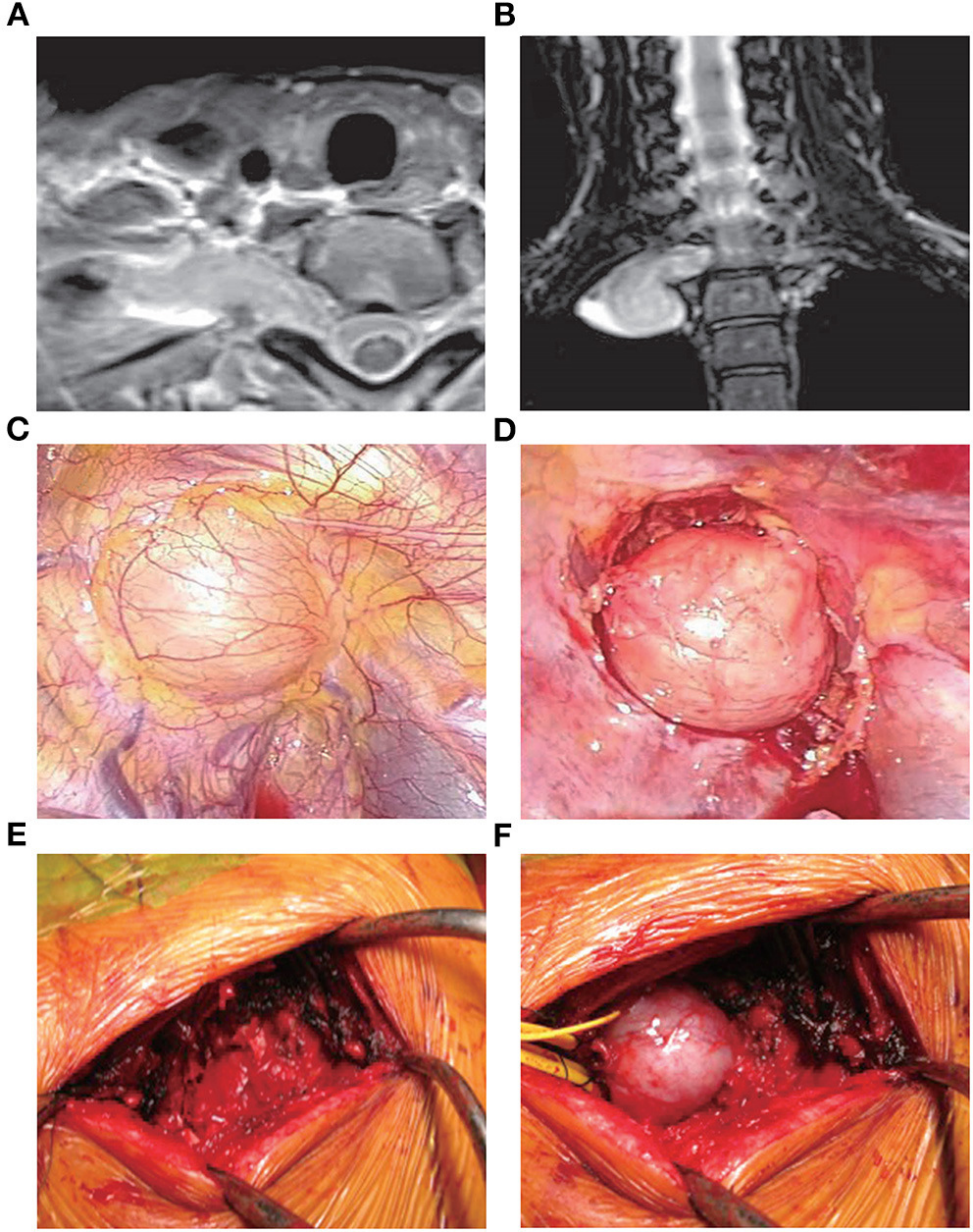
Treatment with VATS (25). A 41-year-old female presented with an abnormal shadow on the apical portion of the right lung and motor weakness of the intrinsic muscle in her right hand. Examination revealed the presence of a dumbbell tumor of the right T1 nerve root, extending to the foramen and paravertebral region. Due to her symptomatic presentation, the patient underwent VATS along with posterior spinal surgery. Initially, anterior release using VATS in the left lateral position was performed. During posterior spinal surgery following VATS, a response to intraoperative nerve stimulation on the T1 nerve root distal to the tumor was confirmed. Therefore, enucleation of the tumor was performed using an operating microscope to preserve the function of the intrinsic muscle. **(A,B)** Magnetic resonance imaging images showing the presence of a dumbbell tumor at the right T1 nerve root, extending to the foramen and paravertebral region. **(C)** The tumor during VATS. **(D)** The tumor after the anterior release of the ambient organs. **(E)** Partial costotransversectomy was performed following VATS. **(F)** The preceding VATS ensured tumor mobility in the prone position. The tumor was moved to the surface through gentle handling. VATS, video-assisted thoracoscopic surgery.

VATS has been widely used in cardiothoracic surgery, and its advantages have been demonstrated (22). The limited damage to the thorax and clear images of the location of the tumors associated with this approach render VATS a suitable option for the treatment of MST. Of note, its advantages become more obvious when applied to the upper thoracic spine (Figure 2) (23, 24).

In 2008, Kan et al. (26) studied five MST patients who underwent tumor excision through a minimally invasive thoracoscopic approach. In all patients, pain and neurological deficits were significantly improved during the 4–6 months of follow-up. However, Huang et al. (27) indicated a 12% rate of excessive bleeding among 41 MST patients treated with thoracoscopic corpectomy. This finding suggests that this approach may be associated with a significant risk of bleeding.

Unfortunately, endoscopic techniques are technically demanding and have not been widely applied in current spine surgery. Thus, the learning curve may be steep. Currently, these techniques are more applicable to the posterior approach for the treatment of MST patients.

### Minimal Access Spinal Surgery

MASS involves smaller incisions and use of an operative microscope to enhance the visual field. This approach is suitable for posterior spinal surgery (28). Endoscopic surgery has shown superiority over MASS in the treatment of MST. However, it is not extensively used due to its high technical difficulties, long duration of surgery, excessive bleeding, and difficulty in achieving hemostasis during the operation. Thus, MASS attracted the attention of surgeons. In MASS, there is no requirement for specialized expensive equipment. This easily mastered technique can safely dissociate the neurovascular structure and reduce the time of the operation (i.e., decompression and stability reconstruction) (29).

Huang et al. (30) compared MASS (29 patients) with standard thoracotomy (17 patients) in the treatment of thoracic MST. The results showed that the therapeutic effects observed in the two groups were similar. Notably, the postoperative rate of stay in the intensive care unit in the MASS and standard thoracotomy groups was 6.9 and 88%, respectively. These findings demonstrated that MASS is beneficial in the treatment of patients with MST, especially in terms of reducing the need for postoperative stay in the intensive care unit. Furthermore, Payer et al. (31) reported a series of 37 patients who underwent minimal access anterior approach corpectomy, including 11 patients with vertebral tumors. The clinical outcomes were favorable with minor hemorrhage and no neurological worsening. Also, Chou et al. (32) illustrated a mini-open way to perform thoracolumbar corpectomy in the aspect of technology. In conclusion, MASS may be a remarkable alternative to thoracoscopic or endoscopic procedures.

### Percutaneous Pedicle Screw Fixation

In open surgery, the traditional approaches for the placement of screws require to separate the surface of the spine from muscle and tissues, leading to extensive tissue trauma and excessive blood loss (33). The development of the PPSF technology has markedly improved the therapeutic effect. This approach is widely used for the treatment of patients with vertebral fractures, without damaging the underlying muscle. The application of X-ray images and guidewires facilitates the efficient placement of screws (34). In addition, PPSF has shown encouraging outcomes in the treatment of pathological fractures caused by metastatic tumors of the spine. This approach—in combination with PVP whenever possible—can simultaneously enhance the stability of the spine and control tumor progression, by fixing the upper and lower one/two vertebral bodies of the fractured centrum (35).

Moussazadeh et al. evaluated 44 consecutive patients with MST who underwent percutaneous spinal fixation through vertebral body cement augmentation. This technology provides the anticipated spinal stability with less blood loss, reasonable rate of complications, and reduced time for patients to return to oncological treatment. The investigators suggested that PPSF is a feasible surgical approach for patients with mechanical instability caused by MST (36). Nevertheless, this technology is unsuitable for patients with long-term survival and complex vertebral instability (37).

As a result, owing to the prolonged survival and the complicacy of vertebral instability in MST patients, available studies investigating the clinical outcomes associated with PPSF are insufficient. Further studies are required to confirm the advantages of this technology. As an important supplement to MIT in the management of MST patients, the long-term clinical application of PPSF—combined with other treatment modalities—is worthy of investigation.

## Minimally Invasive Therapy

Although originally used for the treatment of degenerative spine diseases, at present, MIT is widely used against MST. Patients with MST are suffering from complications, malnutrition, considerable pain, a weakened immune system, and limited life expectancy. For these patients, promoting recovery after surgery and returning to oncological treatment are major objectives. Various forms of MIT have been developed for the management of MST, including cement augmentation techniques, radiofrequency ablation (RFA), percutaneous cryoablation, and implantation of radiant seeds (5, 29, 38).

The MIT techniques have been attached with prompt recovery—through reduction of soft tissue disruption—reduced intraoperative blood loss, and short hospitalization. In addition, they demonstrated encouraging results—comparable with those reported after open surgery—in terms of pain management and neurological improvement (39, 40). Additionally, MIT is linked to a low rate of infection versus open surgery (41). Nevertheless, a recent literature review raised doubts regarding the usefulness of MIT due to inferior quality and the lack of strong recommendations (42).

In contrast, a limited number of researches and investigators continue to support the use of aggressive surgeries (e.g., vertebrectomy), especially in the treatment of thyroid and solitary renal cell spine metastases (43). Moreover, the MIT options for the treatment of cervical spine are limited. Therefore, open surgery remains the gold standard in this setting. Thus, far, only a few technological innovations have attracted considerable attention in this field. Nevertheless, MIT continues to occupy an important position in the treatment of MST, owing to its advantages. Furthermore, treating physicians and patients may also be more inclined to recommend/accept MIT.

### Cement Augmentation Techniques

**Figure 3:**
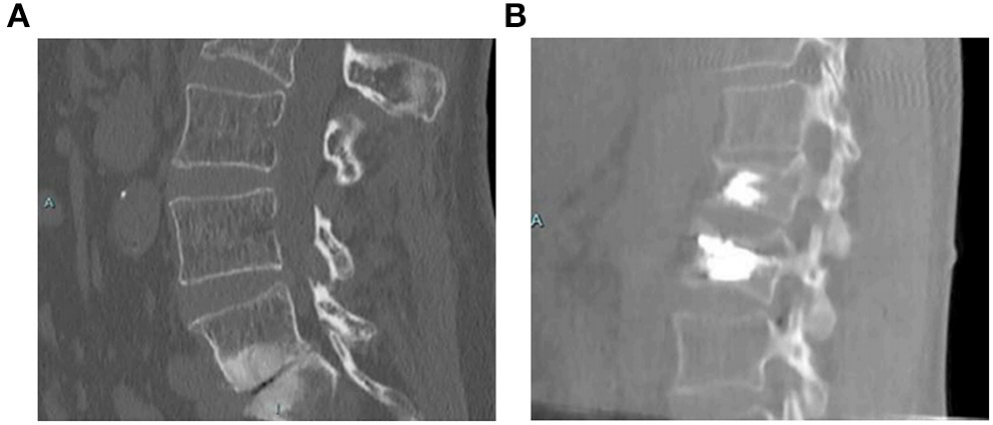
Treatment with PKP (47). A 58-year-old female with a non-specified mitochondrial pathology and a history of L1 vertebral fracture treated with kyphoplasty. She presented with pain in the lumbar region after a fall. She was treated with PKP, and demonstrated an unremarkable postoperative course. **(A)** Computed tomography scan showing a L2 fracture with a deformity in both the sagittal and coronal planes. **(B)** Postoperative scan showing correction in both the sagittal and coronal planes. PKP, percutaneous balloon kyphoplasty.

**Figure 4:**
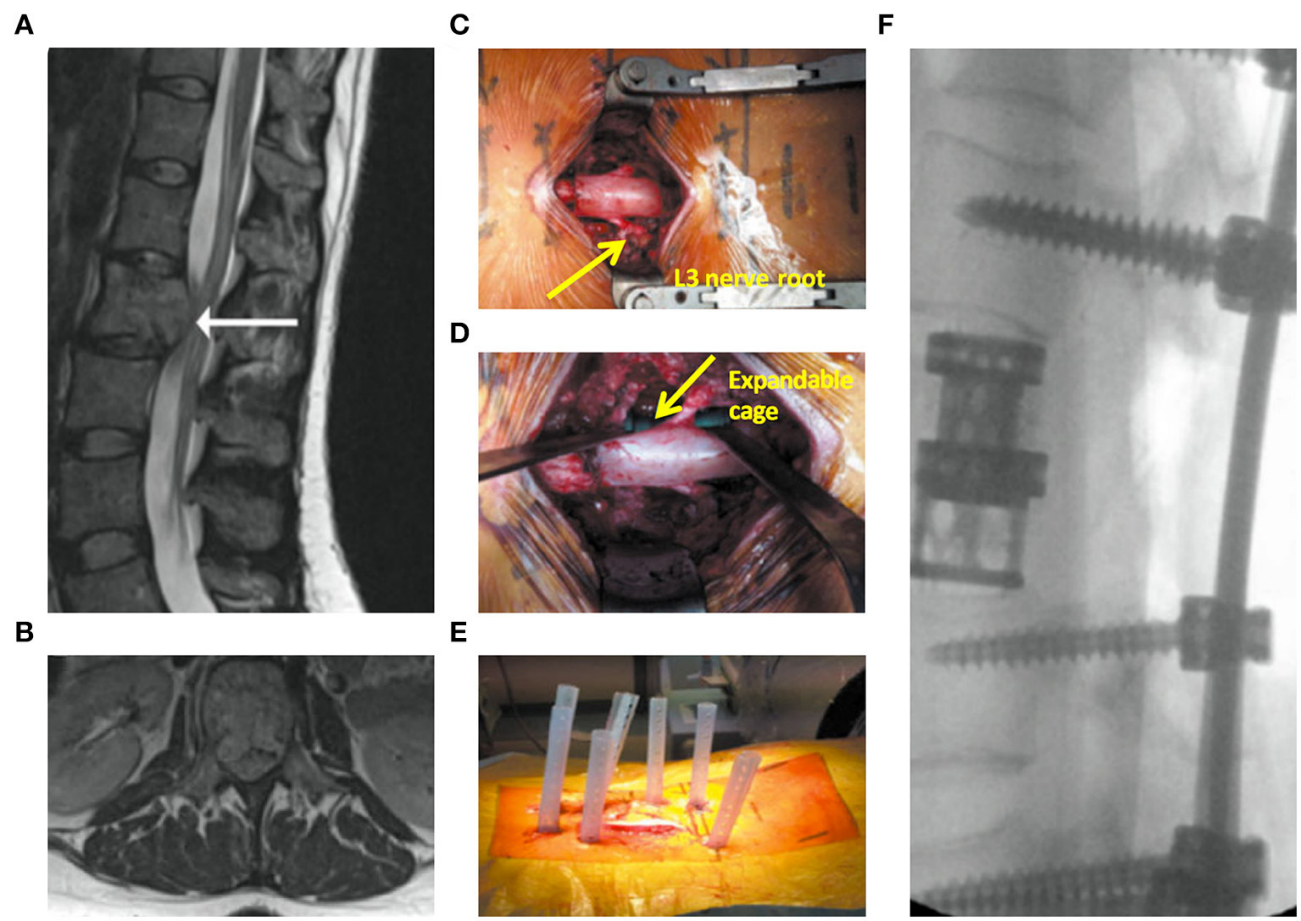
Expandable cage-assisted treatment (39). A 32-year-old female presented with mid-back pain and leg weakness >3 weeks. **(A,B)** Magnetic resonance imaging images showing a solitary L2 lesion causing compression of the circumferential cauda equina. **(C)** Intraoperative photograph showing a mid-line incision with circumferential decompression and vertebrectomy. Decompressed L3 nerve roots are displayed (arrow). **(D)** Intraoperative photograph showing expandable cage (arrow)-assisted reconstruction of the vertebral body. **(E)** Intraoperative photograph showing a mid-line wound closure and percutaneous fixation two levels above and below the vertebrectomy. **(F)** Postoperative radiograph.

Cement augmentation techniques include percutaneous vertebroplasty (PVP) and percutaneous balloon kyphoplasty (PKP) (44). PVP is performed using high-power cement and the force of the poly-methylmethacrylate (PMMA) injection must exceed the local pressure of the cancellous bone of the vertebral body (45). Constant observation is required to prevent leakage of the bone cement. PKP was designed to raise the endplate by introducing an inflatable balloon into the compressed vertebral body (Figure 3). This is achieved by creating a low-pressure chamber filled with cement inside the vertebral body. Recently, insertion of an expandable cage has also shown satisfactory outcomes in MST patients (Figure 4) (46).

Pain alleviation is the primary goal of cement augmentation techniques. The mechanism of pain reduction includes chemical toxicity, a thermal necrosis effect, and mechanical stability of the fractured vertebral body. At present, it is considered that the stability of the vertebral body mechanics is the main mechanism for vertebral body formation to relieve pain (47, 48). In a review of previous literature, Hadjipavlou et al. showed that the rate of pain relief in vertebral tumors was 75.9–92.5% and 75.6–98.2% for PVP and PKP, respectively (49). Moreover, there was no significant difference between the two approaches in terms of pain reduction. Furthermore, both techniques were shown to effectively prevent the occurrence of complications and extend the lives of patients (50, 51).

Currently, PMMA mixed with an opacifier is the most commonly used cement (52). Advanced techniques, such as radiofrequency-targeted augmentation and implants, have improved the application of PMMA (53, 54). Calcium phosphate cement—as a synthetic bone graft—has recently attracted considerable attention, owing to its numerous advantages, such as osteoconductivity and injectability. In addition, it allows the addition of drugs and active substance to the materials because of its intrinsic porosity and low-temperature solidification reaction (52). Furthermore, a radio-opaque silicon polymer may be used as a substitution to bone cement, offering a longer working time and stiffness (55).

Cement augmentation techniques are widely accepted, owing to their extensive applicability, excellent analgesic effect, and high rate of efficacy. However, their effect on tumor inhibition is limited, and the risk of bone cement leakage leading to impaired neurological function remains high. Therefore, we consider that combination therapy may be the most appropriate approach to overcome these challenges.

### Radiofrequency Ablation

In the previous 5–10 years, RFA has been used as an alternative in the palliative treatment of MST (56). By placing the radio-frequency electrode into the tumor, this technology produces thermal damage to the tumor tissue around the electrode, resulting in the destruction of tumor cells through coagulation necrosis (57, 58).

Although RFA can effectively alleviate pain in patients with MST, it cannot improve nerve function or prevent pathological fractures. Zheng et al. (59) combined RFA with PKP for the treatment of 38 vertebral bodies of 26 patients. The results indicated that all patients exhibited prominent pain relief.

Recently, Rosian et al. (60) accomplished a systematic review involving 583 patients from nine studies to assess the clinical efficacy and safety of RFA—in most cases combined with vertebroplasty (*n* = 437)—in patients with MST. The results indicated that all patients exhibited statistically significant pain relief at 1 and 3 months. The purpose of this treatment is palliative; thus, only two studies have reported data related to mortality. Additionally, there were no major complications observed following treatment in any of the nine studies. This evidence demonstrates that RFA plays a significant role in pain reduction and improvement in quality of life.

Moreover, RFA is an alternative treatment that can effectively relieve pain in patients with metastatic vertebral tumors who are not eligible to receive radiation therapy or chemotherapy. However, the limited evidence currently available renders the assessment of RFA applicability difficult. Subsequent studies should investigate the amount of bone cement, improve the accuracy of the injector position, and exercise strict control over the inclusion criteria to further determine the efficacy and safety of the RFA combination therapy.

## Radiation Therapy

The previous decades have witnessed advancements in the development of radiation therapy for the treatment of patients with MST (61). Despite the significant progress in surgical treatment and chemotherapy, radiation therapy remains the basis for the treatment of MST. Radiotherapy has demonstrated a significant effect on pain relief, local tumor control, and neurological recovery in numerous studies (62–64).

With the development of new technology and devices, the treatment of MST through radiation therapy has also evolved. Use of conventional external beam radiation therapy (CEBRT) was gradually abolished due to its limitations. Spinal stereotactic radiosurgery (SSRS) and brachytherapy are recent advances in the management of patients with MST. These techniques safely provide higher radiation doses and currently predominate this setting.

### Conventional External Beam Radiation Therapy

Previously, CEBRT was a major treatment option for spinal tumors. This approach delivers radiation commonly in a dose of 30 Gy over 10 fractions (65, 66). It is reported that highly radiosensitive tumors (e.g., lymphoma and myeloma) and intermediate radiosensitive tumors (e.g., breast, prostate, and lung) are sensitive to CEBRT. However, certain solid tumors (e.g., hepatocellular carcinoma, renal cell carcinoma, sarcoma, and melanoma) are non-responsive to CEBRT (66, 67).

For the reduction of potential treatment errors, the radiation field of CEBRT should include normal tissues around the target area, usually in the range of 1–2 cm. This results in a limited dosage and the occurrence of toxicity in surrounding tissues (68). This led to the development of intensity-modulated radiation therapy, whose model of radiation delivery is determined by critically performing optimization and treatment simulation. Thus, the maximum radiation dose is focused on the target area, while reducing the dose administered to the spinal cord. Nevertheless, the sensitivity of the spinal cord to radiation continues to restrict the safe dose delivered through this technology (69).

### Spinal Stereotactic Radiosurgery

SSRS is a recently developed radiotherapy approach, which is gradually becoming a superior therapeutic method to CEBRT for the treatment of patients with MST. The highly conformal hypofractionated external beam radiotherapy—combined with real-time imaging guidance—allows the delivery of radiation with extreme precision and accuracy, while simultaneously guaranteeing exposure of the surrounding tissues to a safe dose, even for targets located close to the spinal cord (70).

Research studies have shown that irradiation of tumors treated with a high dose per fraction (i.e., 10 Gy/fraction) may kill tumor cells and destroy newly formed tumor vasculatures, which are particularly sensitive to ionizing radiation. This indicates that the ability of SSRS to deliver high-dose irradiation results in extensive tumor cell death (71). Furthermore, the low number of fractions required for the delivery of the dose in SSRS (i.e., 1–3 fractions vs. 10–20 fractions for CEBRT) has been linked to improved patient compliance.

The advantages of SSRS has led to the development of “separation surgery,” which requires circumferential decompression of the epidural tumor to form an interval between the tumor and spinal cord (72). Laufer et al. performed a retrospective review of 186 patients, and the results showed that separation surgery followed by adjuvant SSRS to the remaining tumor is applicable to the control of durable local tumors. In this series of patients, after 1 year, the rate of local tumor progression varied (4–22%), depending on the postoperative dose of radiation. In addition, there was no significant difference found in the rate of local progression between single-fraction and low-dose hypofractionated SSRS (73).

A summary of studies which evaluated SSRS for the treatment of MST is provided in Table 1. Although recent studies have shown that SSRS achieves durable clinical benefits and high local-control rates, current research is confined to retrospective analyses of prospective databases. There is a lack of randomized controlled trials to demonstrate the superiority of SSRS over other methods in the treatment of MST and advance the process of standardization (82). Additionally, further studies are warranted to optimize the dose application standard for SSRS.

**Table 1:** Selected studies of spine stereotactic radiosurgery in treatment of metastatic spinal tumors.

**References**	**Design**	**Details**	**Radiation dose in Gy (range)/fractions (range)**	**Neurological outcome**	**Tumor control**	**Pain relief**	**Overall survival**
Gerszten et al. (74)	Prospective	500 patients, including 344 patients who were previously irradiated	20 (12.5–25)/1	Neurological Improvement: 84%	90%	Long-term: 86%	–
Ryu et al. (75)	Prospective	62 patients 85 lesions	16 (12–20)/1	Neurological Improvement: 81%	1 year: 80%	–	–
Garg et al. (76)	Prospective	61 patients 63 lesions	30/5, 27/3	Neurological deterioration: 13%	1 year: 76%	–	1 year: 76%
Chang et al. (77)	Retrospective	49 patients treated with SSRS reirradiation 93 newly developed patients	20.6/1 19.9/1	–	1 year: 81% 1 year: 89%	1 year: 80.8% 1 year: 89.2%	–
Ahmed et al. (78)	Prospective	66 patients 85 lesions	24 (10–40)/3(1–5)	–	1 year: 89.2%	–	1 year: 52.2%
Laufer et al. (73)	Retrospective	186 patients treated with post-operative SSRS	24/1, (24–30)/3, (18–36)/(5–6)	–	1 year: 83.6%	–	7.1 months: 26.3%
Thibault et al. (79)	Prospective	40 patients treated with SSRS reirradiation 56 lesions	30 (20–35)/4 (2–5)	–	1 year: 73%	–	–
Moussazadeh et al. (80)	Retrospective	31 patients 36 lesions	24/1	Neurological deterioration: 22.2%	5 year: 91.6%	–	–
Ito et al. (81) 2018	Retrospective	131 patients 131 lesions	24/2	–	1 year: 72.3%	1 year: 61.7%	–

### Brachytherapy

By implanting the source of radiation directly to the tumor site, brachytherapy can deliver extremely high doses of radiation while sparing normal tissue. In recent years, advances in radiation therapy have improved the options and applications of brachytherapy (83). Spinal brachytherapy is commonly used as an alternative treatment for patients who are intolerant to other treatments or as an adjuvant treatment combined with other therapies (84). The computed tomography-guided ^125^I seed implantation treatment, which has shown favorable clinical outcomes, is the most frequently used therapy for MST (85).

Recently, Zuckerman et al. (84) performed a systematic review involving 370 patients with spinal metastatic diseases from 15 studies. In these studies, brachytherapy was used to treat patients who had failed previous therapies and were intolerant to open surgery or further treatment. All studies that evaluated pain scores reported a significant improvement in pain control following brachytherapy. Furthermore, few complications were reported.

Spinal brachytherapy is an option for the treatment of MST owing to its advantages (i.e., pain relief, improvement of neurological function, and optimization of local tumor control). However, further studies investigating this prominent procedure should be performed to reach a definitive conclusion.

## Systemic Therapy

Systemic therapy is used to attenuate symptoms caused by spinal tumors. It consists of hormonal therapy, chemotherapy, and the administration of drugs (e.g., corticosteroids, bisphosphonates, and denosumab). The selection of drugs depends on the clinical symptoms of the patients and the type of tumor.

### Hormonal Therapy

Typically, most prostate and breast metastatic tumors are sensitive to hormonal therapy. Although not directly cytotoxic, hormonal blockade exerts an inhibitory effect in the progression of tumors. Use of estrogen receptor inhibitors (e.g., tamoxifen) and selective estrogen receptor downregulators (e.g., fulvestrant) is feasible for the treatment of breast cancer (86). With regard to prostate cancer, androgen deprivation therapy is widely accepted as the palliative treatment of choice (87).

### Chemotherapy

The stability control of spine metastases relies on systemic chemotherapy, which can be performed as monotherapy or combination therapy. This option is characterized by delayed efficacy and limited function. Therefore, it is most commonly used as adjuvant therapy in patients with MST or as an alternative option following failure of initial hormonal therapy (88). Chemotherapy may be the treatment of choice for patients with non-adjacent metastatic spread areas or those not eligible to undergo surgery due to other complications.

Encouragingly, the establishment of cancer immunotherapy has revolutionized the paradigm of cancer treatment, designing to activating and promote the inherent immunological systems to suppress the tumor indirectly. With fewer off-target effects and excellent application potential, cancer immunotherapy alleviates the dependence of tumor treatment on traditional therapies like surgery, chemotherapy, and radiotherapy (89). The immunotherapy for cancer has five major classes: immune checkpoint blockade (ICB) therapy, lymphocyte-promoting cytokine therapy, adoptive T cell therapy, agonistic antibodies against co-stimulatory receptors, and cancer vaccines (89). ICB therapy is the most thoroughly studied immunotherapy category by far (90). Immune checkpoint molecules of cytotoxic T-lymphocyte antigen-4 (CTLA-4) and programmed cell death protein 1 (PD-1) have achieved particular focus for their powerful immunomodulatory effects through acting as negative regulators of T cell activation.

Although significant progress has made in immunotherapy, there are still many challenges before the clinical translation of those innovative therapies, such as the limited therapeutic effect and the uncertain application dose. To realize the application of these therapeutic agents in a safer and more controllable way, new strategies for cancer immunotherapy are urgently needed. Recently, various of biomaterials including nanoparticles, implantable biomaterial scaffolds, injectable biomaterial scaffolds have been developed to improve immune response and anti-tumor effect (91). Delivery strategies utilizing these materials are constantly evolving to trigger powerful therapeutic immune responses with less systemic toxicity (89). In particular, many studies have demonstrated that combination of immunotherapy with other traditional therapies, including chemotherapy, phototherapy and radiotherapy, can produce synergistic effect and improved therapeutic efficacy of malignancies (92). The enhanced immune response generally achieved by immunogenic cell death (ICD) induced by traditional therapies. Besides, many biomaterials that can directly induce immune responses have also been widely studied and achieved promising clinical effects.

### Corticosteroids

Corticosteroids are the mainstay of adjunctive therapy for patients with pain related to vertebral metastases and acute neurodegeneration. The ability of corticosteroids to alleviate pain and spinal cord edema is attributed to their anti-inflammatory effects (93). The clinical effects of these agents have been demonstrated in experimental animal studies, indicating significant improvements in motor function (94).

However, neurological improvements are only observed in the first 2 weeks of treatment with corticosteroids. Therefore, their long-term benefits are insufficient. Moreover, treatment with high-dose corticosteroids is commonly associated with serious systemic side effects (i.e., peripheral edema, hyperglycemia, infections, proximal myopathy, and gastritis). Owing to the lack of improvement in survival and the development of severe side effects, current guidelines have not included the use corticosteroids in the treatment of MST (95).

In the future, the use of nanotechnology may reduce the occurrence of adverse effects while maintaining the curative effect. Hence, further research is warranted to assess the optimal dose of corticosteroids in this setting.

### Bisphosphonates and Denosumab

Bisphosphonates are widely used for the treatment of hypercalcemia and prevention of complications related to metastatic spine tumors, such as pathologic fractures and spinal cord compression. By attaching to the bone surface, bisphosphonates inhibit the activity of osteoclasts, resulting in the reduction of bone absorption and tumor-associated osteolysis. Additionally, bisphosphonates have been shown to possess anti-angiogenesis and anti-tumor effects (96, 97).

A recent study performed by Wilson et al. noted that the incidence of fractures and the time of the first fracture were markedly improved in breast cancer patients treated with zoledronate—a third-generation bisphosphonate. Moreover, the 5-years rate of fracture in patients was reduced to a normal level (98).

Denosumab is a monoclonal antibody for the receptor activator of NF-κB ligand (RANK-L), which plays an significant part in the formation and differentiation of osteoclasts. Therefore, denosumab prevents the combination of RANKL and the receptor activator of NF-κB (RANK), inhibiting the destruction of the bone (99).

Recently, Zhang et al. performed a comparative study showing that denosumab offers advantages vs. zoledronate in decreasing the incidence of skeletal-related events in patients with bone metastases. Furthermore, the subcutaneous injection of denosumab is superior to the intravenous administration of zoledronate in terms of renal toxicity (100).

## Conclusion

MST continue to negatively affect patient quality of life. The current mainstays of treatment for MST include surgical management, MIT, radiation therapy, and systemic therapy.

For surgical management, in spite of the disadvantages, such as the spinal instability and poor surgical outcome of open surgery, it showed a clear advantage in improving ambulatory of patients. Satisfactory prognosis after open surgery depends on the combination with radiation therapy and other treatments. Besides, with the evolution of endoscopic techniques and spinal fixation devices, VATS, MASS, and PPSF have achieved durable clinical benefits. Nevertheless, currently, the surgical field of VATS remains circumscribed. Furthermore, this technically demanding technique involves a steep learning curve and is associated with a significant risk of bleeding. It is thought that the safety of this technique will improve with the development of surgical techniques and instruments. With respect to PPSF, available studies investigating the combination of radiation therapy, cement augmentation techniques with it are insufficient. Further studies are needed to support the advantages of this technology.

For MIT, developments in bone cement materials, RFA equipment have enabled surgeons to more adequately decompress the spinal cord and enhance the stability of the spine, resulting in encouraging clinical outcomes. Thus, in the previous few decades, a transition in the treatment of patients with MST from aggressive open surgery (i.e., spondylectomy) to MIT has been observed. However, currently, this approach is characterized by numerous limitations. With regard to cement augmentation techniques, the inhibition of tumors continues to be limited, while the risk of bone cement leakage is high. Use of combination therapy may be the preferred choice to overcome this problem. Regarding RFA—an alternative treatment for pain relief—the limited evidence currently available renders the assessment of its applicability difficult. It is only one of the first choices for management of primary liver cancer at present. Further studies are required to determine the appropriate dosage of bone cement, accuracy of the injector position, and inclusion criteria. Furthermore, successful surgeries require substantial X-ray radiation to ensure accurate positioning, resulting in damage to the patients and surgeons. Thus, advanced equipment/devices, (i.e., O-arm technology and intraoperative computed tomography) are necessary to improve accuracy and reduce the damage caused by radiation.

Based on the improvement of imaging techniques and focused radiation therapies, SSRS has revolutionized the treatment of MST. However, radioresistance remains an obstacle in realizing permanent tumor control. Moreover, the high risk of vertebral compression fractures accompanied by a large dose per fraction ought not to be overlooked. Although recent studies have shown that SSRS achieves durable clinical benefits, further research is warranted to determine the optimal population and optimize the dose application standard.

In addition, innovations in systemic therapy (e.g., denosumab) have contributed to the amelioration of cancer care, improving local control and reducing skeletal-related events. However, the potential for clinical application is limited by the development of severe adverse effects and resistance to drugs. In the future, exploring novel immunotherapies with biomaterials is a promising and appealing research field. Despite the many obstacles, we could foresee profound clinical improvement can bring benefit to human health in the near future. Besides, the use of nanotechnology may also assist in reducing the occurrence of adverse effects, while maintaining the curative effect.

The enhanced systemic control of tumors, the availability of various surgical treatment options, and the development of innovative treatment modalities have made more choices for patients. In addition, patients with MST are characterized by intricate symptoms or treatment challenges requiring comprehensive and multidisciplinary management. Extra efforts are required for the provision of appropriate care in the management of these patients. Overall, successful treatment relies on the combination of surgical management, MIT, radiation therapy, and systemic therapy. With sustained endeavors and combination therapy, the quality of life of patients with MST will continue to improve.

## Author Contributions

JL wrote the manuscript, curated the data, and searched the literature. CF performed the conceptualization, formal analysis, and supervision. WW, FX, YW, and YL contributed to manuscript writing and figures. All the authors contributed substantially to the final manuscript and approved the final version.

## Conflict of Interest

The authors declare that the research was conducted in the absence of any commercial or financial relationships that could be construed as a potential conflict of interest.

## Abbreviations

CEBRT: conventional external beam radiation therapy
MASS: minimal access spinal surgery
MIT: minimally invasive therapy
MST: metastatic spinal tumors
PKP: percutaneous balloon kyphoplasty
PMMA: poly-methylmethacrylate
PPSF: percutaneous pedicle screw fixation
PVP: percutaneous vertebroplasty
RANK: receptor activator of NF-κB
RANK-L: receptor activator of NF-κB ligand
RFA: radiofrequency ablation
SSRS: spine stereotactic radiosurgery
VATS: video-assisted thoracoscopic surgery.
